# Quantification of fungal biomass in mycelium composites made from diverse biogenic side streams

**DOI:** 10.1186/s40694-024-00189-y

**Published:** 2024-11-18

**Authors:** Marcello Nussbaumer, Tanja Karl, J. Philipp Benz

**Affiliations:** Professorship of Fungal Biotechnology in Wood Science, Holzforschung Munich, TUM School of Life Sciences, Technical University of Munich, Hans-Carl-Von-Carlowitz-Platz 2, 85354 Freising, Germany

**Keywords:** Mycelium composites, Biogenic residues, Mycelium quantification, Real-time PCR, *Ganoderma sessile*, *Pleurotus pulmonarius*, *Trametes versicolor*

## Abstract

Mycelium composite materials are comprised of renewable organic substrates interconnected by fungal mycelium, allowing full biodegradability after use. Due to their promising material properties, adaptability, and sustainable nature, these biomaterials are investigated intensively. However, one crucial aspect that has hardly been covered so far is the proportion of fungal biomass in the composites, which would be necessary to assess its contribution to the material characteristics. Since a complete physical separation of mycelium and substrate is not feasible, we approached this issue by isolating the fungal DNA and relating it to the mass of mycelium with the help of quantitative PCR. Overall, 20 different combinations of fungi and biogenic side streams were evaluated for their handling stability, and growth observations were related to the quantification results. *Ganoderma sessile* was able to form stable composites with almost all substrates, and a positive correlation between mycelial biomass and composite stability could be found. However, the amount of mycelium required for fabricating firm materials strongly depends on the combination of substrate and fungal species used. Less than five mass percent of fungal biomass can suffice to achieve this, as for example when combining *Trametes versicolor* with sugar beet pulp, whereas a mass fraction of twenty percent leads to crumbly materials when using *Pleurotus pulmonarius* on green waste. These results indicate that the mycelial biomass is an important factor for the composite’s stability but that the properties of the fungal hyphae, as well as those of the substrate, are also relevant. The presented quantification method not only allows to estimate fungal growth during composite production but can also improve our understanding of how the mycelium influences the material.

**Supplementary Information:**

The online version contains supplementary material available at 10.1186/s40694-024-00189-y.

## Introduction

Material science nowadays aims to reduce the use of limited raw materials that are energy-intensive to fabricate and increase the utilization of renewable or recyclable products in almost all fields of application [1]. Replacing these unsustainable materials, especially fossil-based plastics, is challenging due to their enormous versatility at a low price [2]. Many different alternatives are thus required to substitute this group. Cost competitiveness can be ensured best when using side-streams or residues from other processes. These often come as loose materials which need to be bound and shaped to a final product, as is the case in polymer biocomposites, “green concrete”, or particle boards [3–5]. Fungal mycelium provides the features to manage this without the need for fossil-based substances or CO_2_-intensive processing [6].

Mycelium composites have been predominantly made from agricultural and forestry by-products so far [7]. Potential applications, including packaging, thermal insulation, acoustic absorption, and architectural design, could be better tailored by testing more possible combinations of fungi and substrates [8–11]. Investigating the suitability of industrial organic side streams is especially interesting when considering the quantitative availability. For example, despite its abundance and similar composition to more established substrates, green waste has not yet been used. More than 75% of the mobilizable technical potential of German biomass is attributed to this side stream [12]. The biomass with the highest total technical potential in Germany is waste paper, of which a large fraction nevertheless is in material use already [12, 13]. Furthermore, the food and beverage industry provide a large source of organic side streams such as sugar beet pulp, fruit juice pomace, or spent grains [7, 14, 15]. These biomass streams often end up in biogas plants where only part of the organic matter is converted [16, 17]. Consequently, the digested biogas substrate could be an interesting substrate for mycelium composites as well. When looking at large waste streams, textile waste is also worth mentioning. Wagner et al. [18] estimate the amount of collected old clothes to 1.0 Mio. t in Germany for 2018 alone. Although the fraction of synthetic fibers was rising in the last decades, natural fibers and especially cotton still play an important role in this waste stream [19, 20].

Utilization of different substrates increases the versatility of mycelium composites, but not all organic substrates will be equally suitable for the fabrication of these biomaterials. The success depends on the characteristics of the substrate, the cross-linking between fungal hyphae and substrate, and the growth of the mycelium—which is determined by the species used and potential supplements [21–23]. Mycelium growth is a widespread selection criterion for fungal species in mycelium composites [24, 25]. However, the majority of recent research articles estimated this parameter only based on surface growth [9, 26, 27] or by using standard media instead of the actual substrates [22, 28]. Quantification of the growth rate does not necessarily correlate with fungal biomass production and the density of the mycelium was, if at all, only assessed qualitatively [9, 26]. Moreover, growth on the substrate surface is usually higher due to increased oxygen availability, potentially leading to an overestimation of the mycelium content of the material [29, 30]. Quantifying fungal biomass in mycelium composites is the best way to gain knowledge of its role in these materials.

This study focused on the quantification of fungal biomass production of three different fungi on eight agricultural, forestry, and industrial by-products and how this property relates to the handling stability of resulting composites. The three fungal strains of *Ganoderma sessile*, *Pleurotus pulmonarius*, and *Trametes versicolor* were selected for this study based on their fast growth and the frequent use of these genera for the fabrication of mycelium composites [7, 31]. A method based on quantitative PCR was established to be able to differentiate between mycelium and substrate and relate the DNA amount to a mass fraction of fungal biomass.

## Materials and methods

### Strain and substrate selection

The wild-type strains of *Ganoderma sessile* (GS) and *Pleurotus pulmonarius* (PP) originated from the stock of the Technical University of Dresden—IHI Zittau (stock numbers 688 and 685, respectively) from fruiting bodies found in Kentucky (USA) and Saxony (Germany), respectively. *Trametes versicolor* (TV) was isolated from a fruiting body collected in Bavaria (Germany).

For the substrates, the focus was on biogenic materials with little value that have not yet been extensively described for their utilization in mycelium composites. Frozen apple pomace (AP) was provided by Mainfrucht GmbH & Co. KG (Gochsheim, Germany). Textile waste from 100% cotton was collected, buttons and seams were removed, and the textile was blended in a kitchen blender (MMB6172SN, Bosch, Gerlingen-Schillerhöhe, Germany) until obtaining cotton fibers (CF). Digested biogas substrate (DBS) and chopped green waste (GW) were collected from Eggertshof Verwertung GmbH (Freising, Germany) in January 2023. The latter one was sieved in a sieve shaker (AS 200, Retsch GmbH, Haan, Germany) for 5 min at 1.2 mm/g and the fraction with a particle size of 1 to 5 mm was used. UPM Ettringen (Ettringen, Germany) provided the paper sludge (PS) from graphic paper fibers (ash content at 950 °C: 45%). Spent brewer’s grains (SBG) were collected from Bayerische Staatsbrauerei Weihenstephan (Freising, Germany) and molassed sugar beet pulp (SBP) originated from Südzucker AG (Mannheim, Germany). These novel substrates are compared to beech sawdust (B) (Räuchergold^®^ HB 500–1000, J. Rettenmaier & Söhne GmbH + Co KG, Rosenberg, Germany), which is readily used for fabricating mycelium composites [31].

### Pre-cultures and substrate inoculation

All media, including potato dextrose agar (PDA), potato dextrose yeast (PDY), rye grains, and all composite substrates, were autoclaved at 121 °C for 20 min before inoculation. The fungal mycelium was taken from Petri dishes containing PDA stored at 4 °C. Ten agar plugs with a diameter of 8 mm were used to inoculate 100 ml of liquid culture of PDY consisting of 2.4 g of potato extract glucose broth (Carl Roth GmbH + Co. KG, Karlsruhe, Germany) and 0.5 g of yeast extract (Sigma Aldrich, St Louis, USA). The flasks were shaken at 100 rpm for 7 days at 26 °C in the dark in an incubation shaker (New Brunswick Innova 42, Eppendorf SE, Hamburg, Germany). Next, half of the liquid was decanted and the remaining suspension homogenized with an Ultra Turrax^®^ dispersing instrument (TP18/10, IKA^®^-Werke GmbH & Co. KG, Staufen, Germany). These 50 ml were poured into a 1200 ml plastic container (Mycogenetics, Everswinkel, Germany) containing 100 g of soaked rye grains (Heinrichs Agrar GbR, Ingelheim, Germany) and 2 g of gypsum (PUFAS Werk KG, Hann. Münden, Germany). The ingredients were mixed and the container closed with a 0.2 μm microfilter and a screw cap.

Incubation took place at 90% relative humidity (RH) and 26 °C from this step on. After 8 days of growth, all the different substrates were inoculated with 25% (dry mass rye / dry mass substrate) of this grain spawn in plastic bags (SacO2, Deinze, Belgium) which were subsequently sealed with tape. The substrates contained 15 g potato extract glucose broth, 6 g gypsum, and 3 g calcium carbonate (Carl Roth GmbH + Co. KG, Karlsruhe, Germany) per 150 g of dry weight to provide all essential nutrients necessary for the initiation of growth. For the apple pomace, digested biogas substrate, and spent brewer’s grains, the water content was left as it was on delivery (see Table 1). As the high water content (80%) of SBG was inhibiting fungal growth at the bottom, this substrate was also dried and used with 67% water content.

**Table 1:** Substrates with corresponding water contents and each fungus inoculated for composite production

Substrate	Water content in %	Inoculated with		
		GS	PP	TV
Apple pomace (AP)	65	X		
Beech sawdust (B)	67	X	X	X
Cotton fibers (CF)	67	X		
Digested biogas substrate (DBS)	71	X		
Green waste (GW)	67	X	X	X
Paper sludge (PS)	55	X	X	X
Spent brewer’s grains (SBG)	67 and 80	X	X	X
Sugar beet pulp (SBP)	55	X	X	X

The number of biological replicates was n = 5 for each combination except *P. pulmonarius* on paper sludge and sugar beet pulp (n = 4) and *P. pulmonarius* and *T. versicolor* on spent brewer’s grains with 80% water content (n = 3)

After 7 days of colonization, the mycelial network in the substrate bags was disrupted and everything mixed in order to stimulate the formation of a stronger hyphal network with a better distribution within the substrate [32]. This step was repeated two days later and the material was transferred into open Petri dishes in a plastic box directly afterwards. Incubation was terminated after 12 more days by freezing at − 20 °C.

### Estimation of the material’s density, pore volume, and handling stability

The frozen specimens were vacuum-dried in a lyophilizer (Beta 1–8 LMC-1, Martin Christ Gefriertrocknungsanlagen GmbH, Osterode am Harz, Germany) for at least 24 h. Their height (*h*) was measured with a sliding caliper and a cylinder was cut out manually from the center of every specimen with a cork borer (*d* = 20 mm). These cylinders were frozen and lyophilized again before determining their mass (*m*), which was used to calculate the composite’s densities (*ρ*) with Eq. (1).

1 $$\rho = \frac{{\text{m}}}{{\frac{{{\text{d}}^{{2}} \cdot \pi \cdot {\text{ h}}}}{{4}}}}$$

A gas pycnometer (AccuPyc 1330, Micromeritics, Georgia, USA) with helium was used for measuring the particle volume. Subtracting this from the actual volume then gives the pore volume. As helium can access air voids with a diameter down to 0.35 nm, the measured porosity accounts for all open pores in the materials [33].

The handling stability was evaluated with the remaining composite samples after cutting out of the cylinders. The assessment was based on crumbling, crack development, and firmness of the specimens and documented with camera (D780 Body, Nikon corporation, Tokyo, Japan) pictures with a Nikon AF-S 60/2.8 G ED Micro lens.

### Quantification of fungal biomass based on DNA content

#### Growth of mycelium for the relation between DNA and biomass

To evaluate the amount of DNA in a certain mass of fungus, mycelium was grown on beech wood wafers. Similarly to the fabrication of composites, 50 ml of PDY liquid culture were inoculated with five agar plugs (d= 8 mm) for the three fungi. After 7 days at 26 °C and 100 rpm in the incubation shaker, half of the liquid was decanted and the rest was homogenized with the Ultra Turrax^®^. These 25 ml were then evenly distributed to two glass jars (J. Weck GmbH u. Co. KG, Wehr-Öflingen, Germany) containing 25 g of soaked rye grains each and sealed with micropore tape (3 M, Minnesota, USA). After complete colonization, a layer of beech sawdust with 67% water content and a plastic mesh with a mesh size of 2 mm were added on top of the rye. The two incubation steps with rye and beech sawdust took 5 days and 4 days, respectively, for *G. sessile* at 26 °C and 90% relative humidity. For *T. versicolor*, they took 6 days and 5 days, respectively and for *P. pulmonarius*, 7 days and 6 days, respectively. Next, two wafers (50 mm × 20 mm × 10 mm) of beech wood—soaked with water for at least 40 h—were placed onto each plastic mesh and the setup was incubated under the same conditions until the wafers were completely covered by mycelium. This step took 7 days, 10 days, and 12 days for jars with *G. sessile*, *T. versicolor*, and *P. pulmonarius*, respectively. The fresh mycelium was then separated from the wafers with a tweezer and frozen at − 20 °C before lyophilization overnight.

#### DNA extraction

A ball mill (MM 300, Retsch GmbH, Haan, Germany) was used to pulverize the lyophilized mycelia as well as the cut-out cylinders from the composites for 1.5 min at a frequency of 30 s^−1^. The vessels were washed with water and cooled with liquid nitrogen prior to each run. 19 ± 1 mg of the resulting powder was weighed into preweighed 1.5 ml tubes, frozen, lyophilized, and weighed again before starting the DNA extraction. Fungal growth on the wafers was sufficient (> 19 mg) to use mycelium from one wafer per DNA extraction, with the exception of *P. pulmonarius*, where one of the five replicates consisted of mycelium from two wafers.

The protocol of the DNeasy Plant Mini Kit (Qiagen GmbH-Germany, Hilden, Germany) was followed with small adaptions. 400 µl of lysis buffer and 4 µl of RNase A (100 mg/ml) were added to the powders and the tubes were vortexed. During the 90 min of incubation at 65 °C, the tubes were inverted several times every 30 min. After brief centrifugation, 130 µl of buffer P3 were added and the tubes were put on ice for 5 min. For separation of the lysate from the powder and any precipitates, the tubes were centrifuged at 20,000 × *g* for 5 min and the supernatant was pipetted into a QIAshredder spin column. The lysate was then filtered through the column at 20,000 × *g* for 2 min before it was transferred to a new tube. The remaining volume was quantified with a pipette so that 1.5 times this volume of buffer AW1 could be mixed in by pipetting. This mixture was filtered through a DNeasy Mini spin column at 8000 × *g* for 1 min which was then washed twice with 500 µl of buffer AW2. After the second addition of washing buffer, the column was centrifuged at 20,000 × *g* to dry the membrane. The DNA was then eluted into a fresh tube by adding 100 µl of elution buffer onto the membrane and centrifuging it through at 8000 × *g* after 5 min. A second, identical elution step was performed to increase the yield, resulting in a final volume of 200 µl.

#### Fluorometer measurement of DNA extracted from pure mycelium

**Fig. 1:**
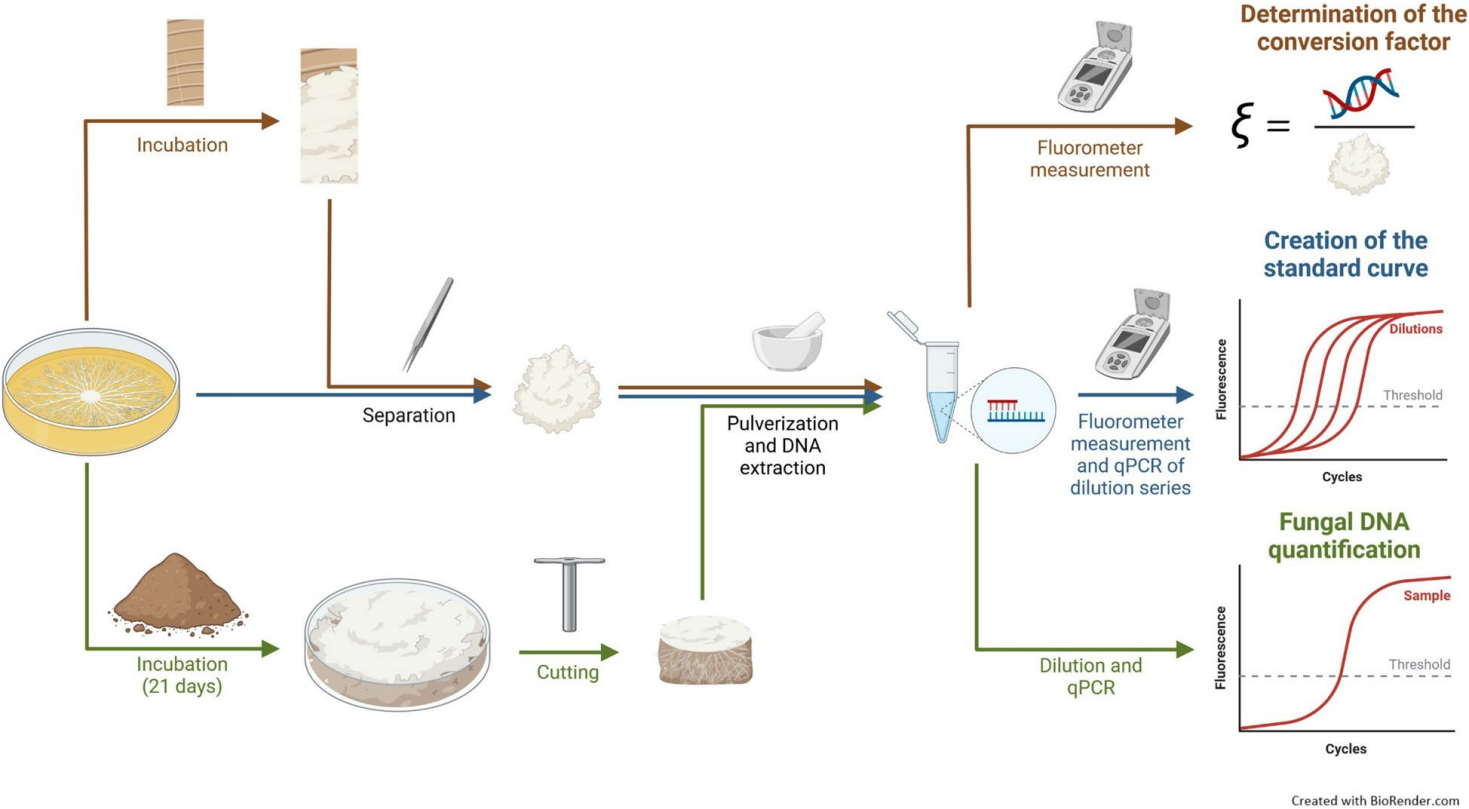
Scheme of the mycelium quantification methodology. Steps include DNA extraction from fungal mycelium grown on beech wafers (brown pathway) and PDA (blue pathway) and measurement of the concentration with a fluorometer. The amount of DNA contained per mass of mycelium from wood wafers was defined as conversion factor *ξ*. DNA extracted from mycelium grown on PDA was used to create a standard curve relating DNA concentration to Cq values. The green pathway shows the composite fabrication, cutting of a representative cylinder for DNA extraction from the center, and qPCR of diluted template samples to quantify fungal DNA based on the standard curve. Finally, the detected amount of fungal DNA was translated to fungal biomass via the conversion factor *ξ*

The 200 µl of DNA solution extracted from pure mycelia were analyzed with a fluorometer (QFX, DeNovix Inc., Delaware, USA). The broad range Denovix dsDNA-Assay was performed according to the manufacturer’s protocol with slight changes. A working solution was prepared containing assay buffer, dye, and enhancer at a volume ratio of 100:1:1. Directly after mixing, 190 µl were transferred into each thin-walled tube, 10 µl of template were added and the mixture vortexed briefly. After 5 min at room temperature, the samples were measured. With a previously measured two-point (0 pg/µl and 200 ng/µl) calibration curve, the relative fluorescence units (RFU) were converted into a concentration. The 200 ng/µl standard was used in every measurement to account for any deviations from the calibration curve by adapting the conversion factor between RFU and concentration. A conversion factor for every fungus (*ξ*_fungus_) was calculated with Eq. (2) using the measured concentration (*ρ*_DNA_), the total volume of DNA solution (*V*_DNA_ = 200 µl), and the mass of mycelium used for the extraction (*m*_m_) to relate DNA to biomass (Fig. 1). This value was based on five replicates for every fungus.

2 $${\xi }_{\text{fungus}} =\frac{{\rho }_{\text{DNA}}\cdot {V}_{\text{DNA}}}{{m}_{\text{m}}}$$

#### Quantitative PCR of DNA extracted from composites

Before quantitative PCR, the DNA concentration was measured on a microplate reader (Infinite 200 Pro, Tecan, Männedorf, Switzerland). All samples were diluted to a concentration of 0.1 ng/µl with nuclease-free water (Thermo Fisher Scientific, Massachusetts, USA) for reasons of comparability and to minimize the effect of PCR inhibitors.

Each tube for qPCR contained 5 µl 2× qPCRBIO SyGreen Mix No-Rox (PCR Biosystems Ltd., London, England), 2.5 µl Primer mix (1.6 pmol/µl of both primers), 1.5 µl nuclease-free water, and 1 µl DNA solution. Every template was measured in triplicate and the mean value used for further calculation. In addition to the DNA solutions extracted from the composites, templates from uncolonized substrates were measured to ensure that there was no background of fungal DNA. Forward and reverse primer for *P. pulmonarius* and *T. versicolor* were selected from literature [34, 35] whereas the primer pair for *G. sessile* (forward: 5′-TTGTAGAGCGTGTCTGTGCC-3′; reverse: 5′-CGATGCGAGAGCCAAGAGAT-3′) was designed with a primer designing tool [36]. The qPCR runs were performed with a magnetic induction cycler (MIC, Bio Molecular Systems, Queensland, Australia) with an initial denaturation phase of 10 min at 95 °C followed by 40 cycles of 15 s at 95 °C and 50 s at 60 °C. A melting curve was created between 60 °C and 95 °C at 0.5 °C/s.

A standard curve was established with DNA extracted from pure mycelium of every fungus grown on PDA (Sect. "DNA extraction"). The concentrations were measured with the fluorometer and the samples diluted to 8 ng/µl. A dilution series (1:10) down to 80 fg/µl was pipetted and qPCR performed as described above. In the case of *P. pulmonarius*, only the four templates with the highest concentrations were used due to deviations between the three technical replicates at lower concentrations.

Logarithmic regression was used to determine the relationship between template concentration and quantification cycle (Cq) (Threshold level: 0.300 of normalized fluorescence). The coefficient of determination was *R*^*2*^ = 0.9997 for *G. sessile*, *R*^*2*^ = 0.9988 for *P. pulmonarius*, and *R*^*2*^ = 0.9996 for *T. versicolor*. This logarithmic relationship allowed for conversion from Cq values to DNA amount after the qPCR of diluted composite DNA solutions. The obtained DNA amount was used as concentration (*ρ*_DNA_) in Eq. (3) as it originated from 1 µl of DNA solution. It was then multiplied with the volume of the total DNA solution after dilution to 0.1 ng/µl (*V*_DNA_) to determine the mass of DNA extracted from the initial composite sample. Relating this mass to that of the composite sample (*m*_c_) provides a mass fraction of DNA, which can be translated to the mass fraction of mycelium (*x*_m_) with the previously introduced conversion factor (*ξ*_fungus_) as in Eq. (3). To relate the mycelial mass to the composite volume instead of its mass, *x*_m_ was multiplied with the density of the corresponding composite sample.

3 $${x}_{\text{m}} =\frac{\frac{{\rho }_{\text{DNA}}\cdot {V}_{\text{DNA}}}{{m}_{\text{c}}}}{{\xi }_{\text{fungus}}}$$

### Statistical analysis

The data was analyzed in RStudio 2023.06.2 [37]. None of the data sets were normal distributed according to the Shapiro–Wilk normality test (p < 0.05). After confirming significant differences with the Kruskal–Wallis test, Dunn’s post-hoc test was used to compare different specimens pairwise with the PMCMRplus package [38]. Only materials containing the same fungus or substrate were compared with each other.

## Results

### Preparation of composites and estimation of the material’s stability

**Fig. 2:**
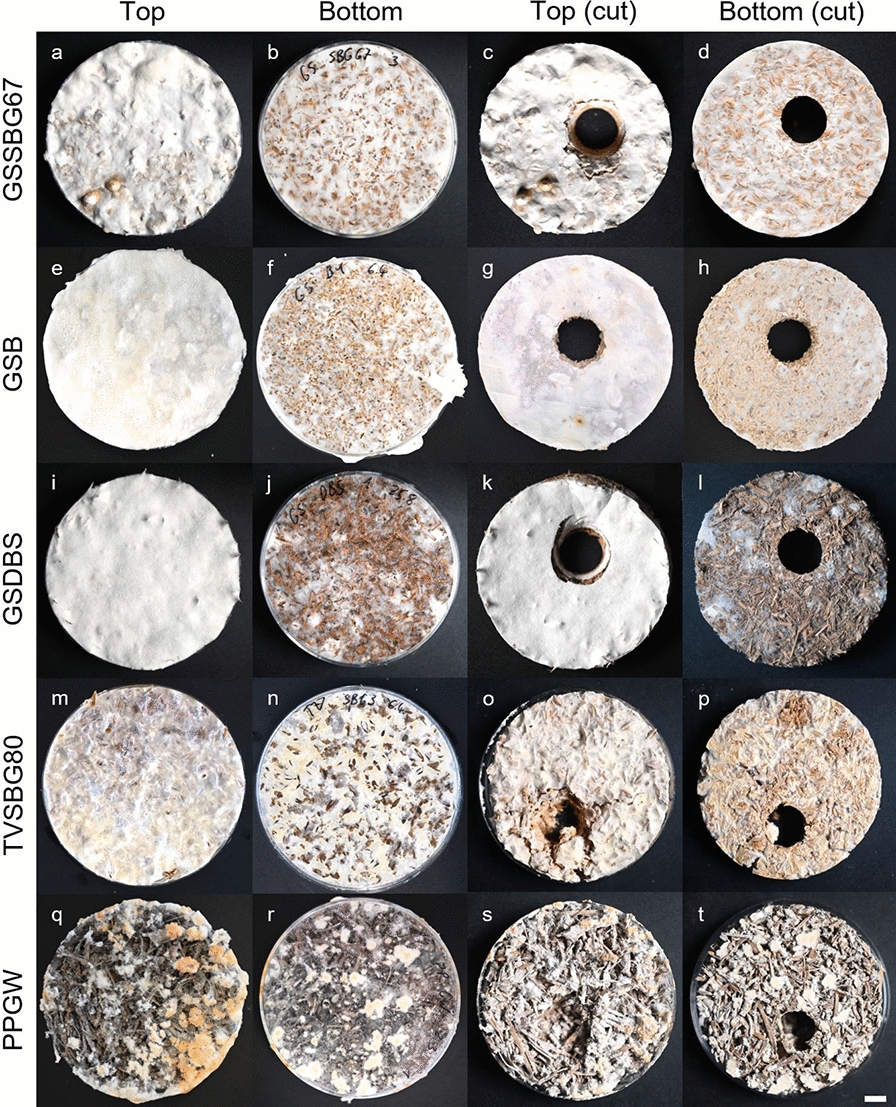
Images of mycelium composites before lyophilization (left half) and dry materials after cutting (right half). Different categories of handling stability 4 (**a**-**d**), 3 (**e**–**h**), 2 (**i**-**l**), 1 (**m**-**p**), and 0 (**q**-**t**) are represented. Scale bar = 1 cm

**Fig. 3:**
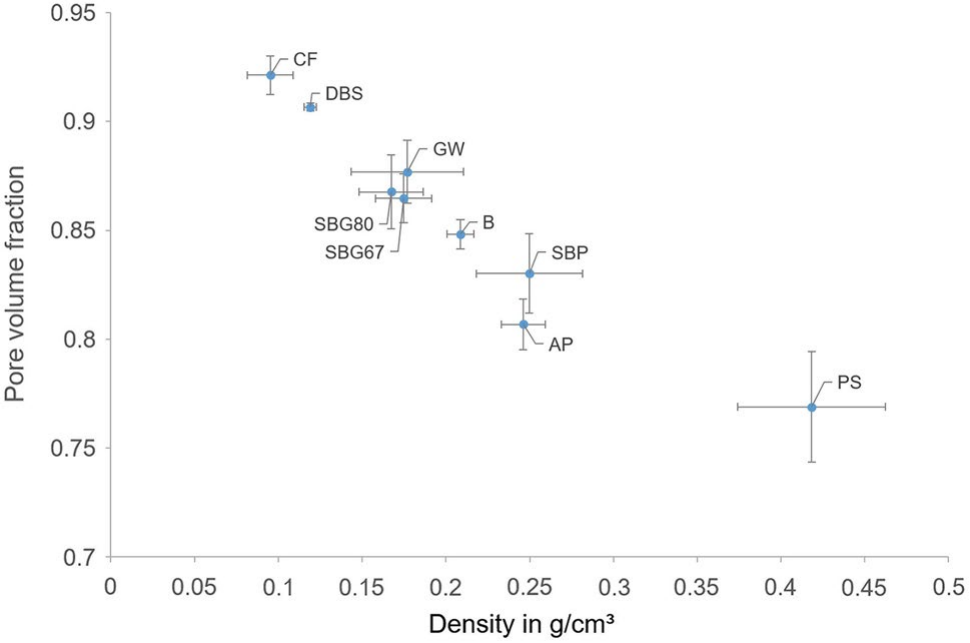
Density and pore volume fraction of the composites made from different substrates. Values represent arithmetic means with standard deviations of all biological replicates of the three fungal species (Table 1)

Mycelium composites were fabricated with different fungi and substrates (Table 1). To rate the suitability of the tested fungus-substrate combinations for composite production, their handling stability was categorized after 21 days of incubation. After cutting out cylinders from the center of the composites, they were inspected and rated into one of five categories (Table 2, Fig. 2). Fungal growth was observed on all of the selected biogenic residues and stable composites could usually be obtained for at least one fungus, demonstrating that the screening of fungal-substrate combinations to take advantage of their species-specific adaptations to different lignocellulosic materials is highly worthwhile. Only for cotton fibers (CF), digested biogas substrate (DBS), and paper sludge (PS), the grown mycelium could not provide sufficient adhesion for a good handling stability. In the case of PS, the formation of fiber clusters with diameters larger than 1 cm caused large gaps between them, making it potentially more difficult for the hyphae to interconnect into firm mycelia. A similar issue might have occurred for DBS and CF due to their high pore volume (Fig. 3).

**Table 2:** Categorization of handling stability of the fabricated composites

Handling stability	Composite materials
Stable material with firm appearance (4)	GSAP, GSSBG67, TVSBP
Stable material with a thinner mycelium layer on the bottom (3)	GSB, GSGW, GSSBG80
Material is fragile (poor adhesion inside); after cutting, substrate particles may fall out (2)	GSCF, GSDBS, GSPS, TVB, TVGW, TVSBG67
Severe cracks develop when cut, which can make the material fall into pieces (1)	PPB, PPPS, TVPS, TVSBG80
Weak adhesion leads to crumbling as soon as the material is demolded (0)	GSSBP, PPGW, PPSBGs, PPSBP

Visual examples are contained in Fig. 2

### Density and pore volume

The density and pore volume fraction of the substrates were in a similar range after inoculation and incubation with the three different fungi. Hence, the average values from all composites with the same substrates were plotted (Fig. 3). There was no significant decrease in density caused by higher fungal growth on a specific substrate (Additional file 1, sheet 1). A clear inverse correlation was observed between density and pore volume of the composites. The lightest substrates (CF, DBS) exceeded a pore volume of 90%.

### Quantification of fungal biomass based on DNA content

After DNA extraction from the composite samples, the amount of fungal DNA was determined using qPCR and previously created standard curves from DNA of pure mycelium (Fig. 1). The amount of fungal DNA could then be converted to mycelial biomass via the conversion factors, which were calculated based on the DNA content of mycelium grown on beech wafers. This lignocellulosic substrate was chosen since its nutrient composition is more similar to those of the composite substrates than PDA, helping to reduce the probability of variations in DNA content per fungal biomass. The conversion factors (*ξ*) are given as arithmetic means with standard deviations of five replicates in microgram of DNA per gram of mycelium: *ξ*_GS_ = 105 ± 19, *ξ*_PP_ = 287 ± 27, and *ξ*_TV_ = 177 ± 31.

To rule out the presence of any background DNA amplification from the pure lignocellulosic substrates, DNA was extracted from these before inoculation with any fungus and qPCRs were performed with the primers of all fungi that were later grown on these substrates. The measured amount of fungal DNA corresponded to a mycelium fraction of below 0.1 m.% in all substrates and was therefore considered neglectable.

The woody/lignocellulosic substrates beech sawdust and green waste led to measurable growth for all three tested fungal strains. The highest production of mycelium was achieved by *G. sessile* on apple pomace (AP), spent brewer’s grains with 80% water content (SBG80), and green waste (GW) with 57 mg/cm^3^, 41 mg/cm^3^, and 37 mg/cm^3^, respectively, and all exceeding 20 m.% of mycelium (Fig. 5). For *P. pulmonarius*, the content of mycelium grown on SBG and sugar beet pulp (SBP) was less than 1 m.% and 1 mg/cm^3^. On beech sawdust (B), this fungus produced the least biomass as well but outperformed *T. versicolor* on GW and PS significantly.

**Fig. 4:**
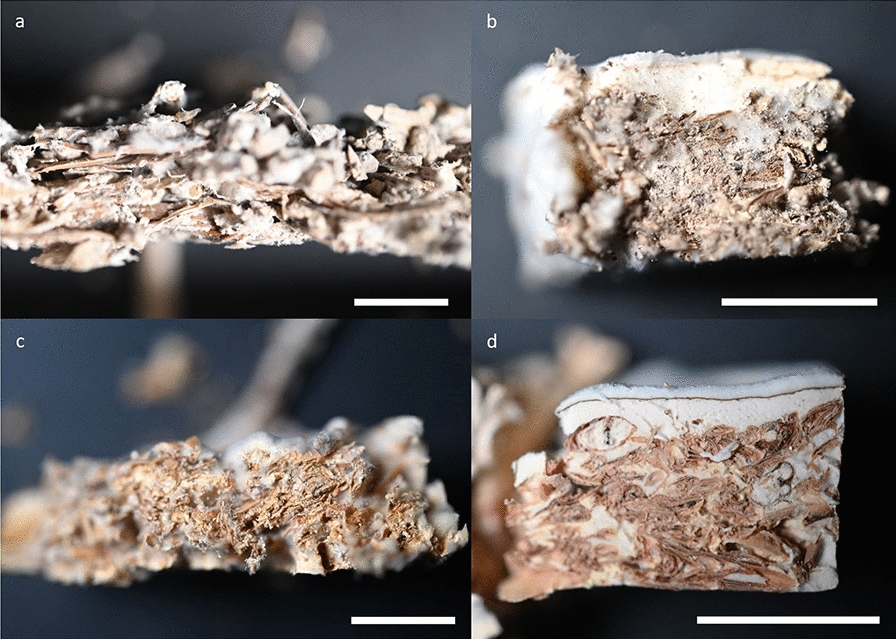
Pictures of composite cross sections from PPGW (**a**), GSGW (**b**), TVSBG67 (**c**), and GSAP (**d**). The mycelium distribution within the materials is shown. Pictures a-c show composites with similar mycelium content for the three different fungi whereas d visualizes dense mycelial growth. Scale bars = 1 cm

Compared to the two other fungi, *G. sessile* produced a continuous layer of aerial mycelium on top of almost all substrates, albeit with varying density (Fig. 4b, d). Interestingly, the biomass production of this fungus on the spent brewer’s grains with higher water content (SBG80) was significantly larger than on the ones with lower water content (SBG67) despite of standing water in the molds. The visible lack of growth on the wet bottom was seemingly compensated by growing more aerial mycelium. Both other fungi were struggling with the high water content, which also led to contamination with mold fungi for some samples (excluded).

**Fig. 5:**
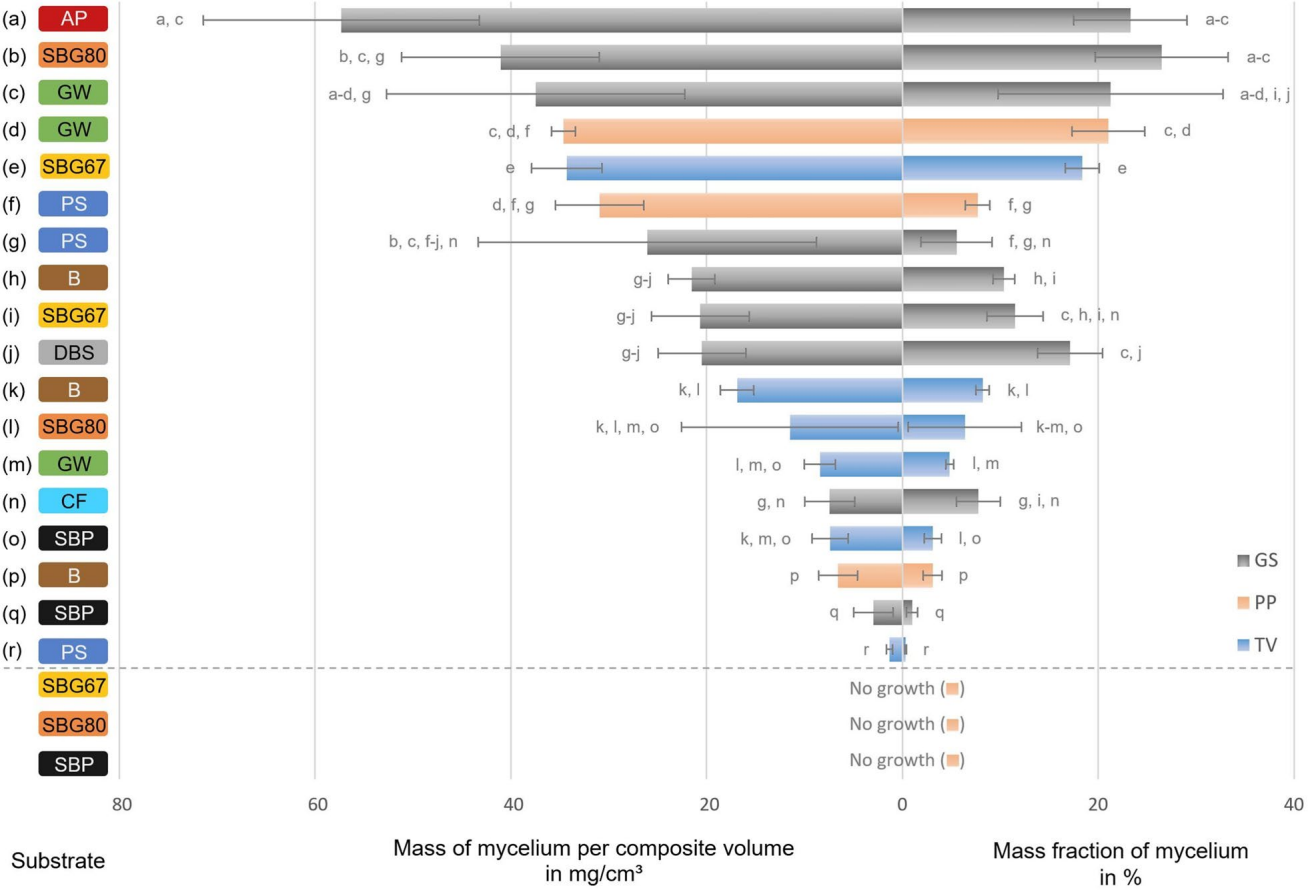
Fungal biomass production during 21 days of incubation of 20 different fungus-substrate combinations. The results are displayed as mycelium mass per composite volume (left) and per composite mass (right). The data were sorted by mycelium mass per composite volume. Composites with low densities rank higher when the mycelial biomass is related to the composite mass instead of its volume and vice versa for dense materials. Values represent arithmetic means with standard deviations of five biological replicates for all combinations except TVSBG80 (n = 3) and PPPS (n = 4). Different letters indicate statistically significant difference to materials containing the same fungus or substrate (Dunn, p ≤ 0.05). (AP: Apple pomace, SBG67/80: Spent brewer’s grains with 67% / 80% water content, GW: Green waste, PS: Paper sludge, B: Beech sawdust, DBS: Digested biogas substrate, CF: Cotton fibers, SBP: Sugar beet pulp, GS: *Ganoderma sessile*, PP: *Pleurotus pulmonarius*, TV: *Trametes versicolor*)

## Discussion

The obtained results indicate that the hyphae of different fungal species contribute differently to the material’s stability (Table 2). The mycelium of *P. pulmonarius* that grew on the substrates within the 21 days of incubation had very weak binding capacity, leading to crumbly specimens. On one hand, the tensile strength of the hyphae—which is known to be species-specific—influences the adhesion [39–41]. On the other hand, cross-linking between hyphae and substrate can also be a limiting factor [22, 23], which depends on the fungal species and the available nutrient sources [42]. Depending on the pore size, a high content of air might also exacerbate the bridging of gaps between substrate particles or fibers for fungal hyphae, causing weak binding inside of the material. This is a potential explanation for the instability of composites produced from CF and DBS. However, testing these two substrates with more fungi than *G. sessile* will be necessary to confirm this issue. A very low pore volume fraction, on the other hand, can reduce oxygen diffusion into the material core and thus lead to lower hyphal densities in mycelium composites [6]. Another parameter that potentially influences the material’s stability is the deactivation process, which is usually oven drying. According to Santos et al. [43] the temperature used for dehydration of mycelium composites can affect their structure, but its effect on the mechanical properties is not conclusive [43, 44]. The vacuum drying procedure we applied might also have had an effect on structure and stability, but was considered more suitable than oven drying for the present study considering the better preservation of DNA at lower temperatures [45]. Besides the adhesion by fungal hyphae, the handling stability of the biomaterials also depends on the density, the particle/fiber size and shape, as well as self-adhesion of the substrate particles [21]. Together with the fact that some fungus-substrate combinations exhibit stronger cross-linking than others, this might explain why composites of *T. versicolor* on sugar beet pulp are more stable than on spent brewer’s grains despite less mycelial biomass (Fig. 5).

**Fig. 6:**
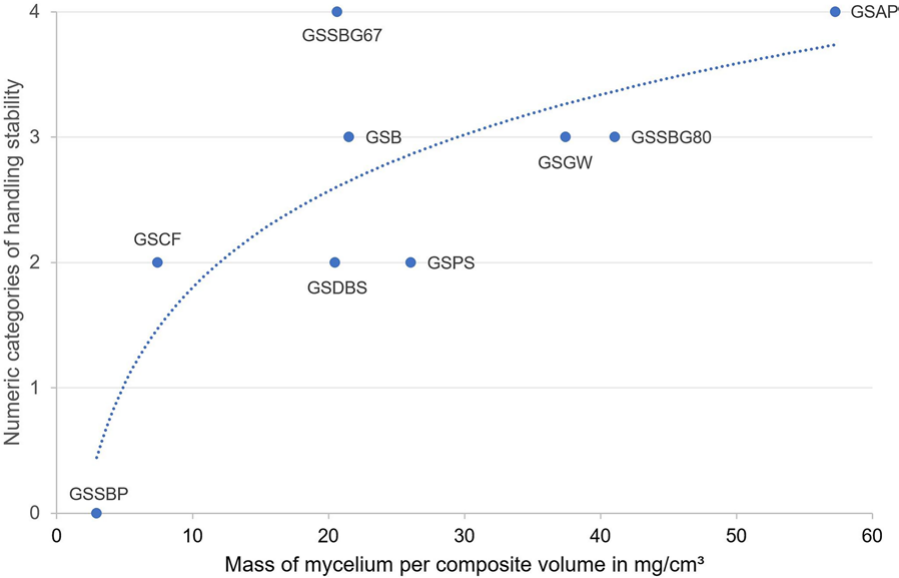
Relationship between handling stability and mycelial biomass following a logarithmic trendline

For *G. sessile*, which was grown on all substrates, a positive correlation was observed between the stability of the composites and the amount of mycelium per composite volume (Fig. 6). It has to be considered that the classification in only few stability categories is relatively rough. An actual mechanical test with a universal testing machine would allow for a better quantification of mechanical properties, but was not possible for the full spectrum of materials due to the instability of some and was therefore not performed. While the trend was nevertheless clear, GSSBG67, GSDBS, and GSPS did not follow the logarithmic trendline, strongly indicating that the nature of the substrate and the mycelium distribution in it also play a decisive role for the overall composite stability. GSPS was more fragile than the logarithmic function would predict, which can be attributed to the large fiber clusters, making it difficult for hyphae to interconnect them. Specimens of GSSBG67 were more stable than composites with a similar mycelium quantity per volume, such as GSB and GSDBS. Most likely, the mycelium distribution favored better handling stability for GSSBG67 because a layer of mycelium covered the whole material, including the bottom (Fig. 2b). The higher water content and smaller water sorption capacity for digested biogas substrate and beech sawdust, respectively, could have led to the accumulation of gravitational water, reducing oxygen supply and thus growth at the bottom (Fig. 2f, j) [46, 47].

A challenging aspect in many studies about mycelium composites is the growth assessment based solely on visual appearance [9, 25, 27, 48, 49]. One reason are different growth characteristics between species, such as aerial mycelium. In the present study, we found composites with *G. sessile* to be covered by a thicker layer of mycelium, potentially leading to the assumption of good colonization. This way, one could mistakenly estimate the degree of colonization of GSGW to be substantially higher than that of PPGW or TVSBG67 (Fig. 4a–c). Another challenge is that the visual distinction between mycelium and substrate cannot account for different mycelial densities quantitatively. On green waste, *G. sessile* grew fluffy, whereas on apple pomace, it was dense (Fig. 4b, d). These observations indicate that it is not sufficient to estimate the mycelium content based on the growth rate on the substrate surface and even the inspection of the cross section can lead to misinterpretations. A more elaborate way of assessing fungal growth is to track the metabolism. This is possible either by quantifying the degradation of the substrate or the CO_2_ production (or O_2_ consumption) during growth [50, 51]. However, these indirect methods do not deliver absolute values of fungal biomass but only a relative growth estimation. Moreover, a comparison between different fungi, substrates, growth conditions, and growth stages might be challenging due to different metabolic activities [50–54].

For understanding the contribution of the mycelium to the material properties, it is crucial to know the mass fraction of fungal biomass to the substrate. Especially when optimizing the incubation conditions and time, this factor can be of utmost importance for minimizing growth duration and production costs while retaining material requirements. Still, only a few publications on mycelium composites tried to estimate this mass fraction so far. Jones et al. [55] and Islam et al. [56] based their quantification on ergosterol, which is only present in fungi, some algae, and protozoa [57]. The concentration was determined by high performance liquid chromatography and converted to fungal biomass via a linear relationship [55, 56]. Islam et al. [56] presented their results in volume percent and did not distinguish between mycelium and pores, leading to a distribution of around 30 vol.% substrate and 70 vol.% mycelium (+ pores) for their composites of mycelium (fungal species not mentioned) and corn stover particles. For the composites of *Trametes versicolor* and *Polyporus brumalis* on wheat straw, rice hulls, and sugarcane bagasse fabricated by Jones et al. [55], the determined content of mycelium was around 5 m.% [42]. Comparing the mycelium quantification of different fungus-substrate combinations is difficult, which is why a comparison with the same materials would be insightful. This is also true when the sensitivity of different quantification methods is of interest. As it is the case for DNA, the ergosterol content in mycelium can vary between different species and depends on growth conditions and time [35, 58]. Additionally, ergosterol has to be handled carefully to avoid chemical or enzymatic losses, which can easily occur [58], and light protection is crucial to avoid photochemical degradation [57]. Studies comparing ergosterol with DNA-based methods for the colonization of wood by basidiomycetes have attributed a higher sensitivity and suitability to the qPCR method, also mentioning the advantage of distinction between targeted species and, for example, molds [35, 59].

Another approach was used by Irbe et al. [60], who attempted to separate mycelium and substrate by grinding and sieving. The mesh size used to separate the fractions was 1 mm and the degree of impurities was determined by microscopy [60]. Depending on the additional nutrient sources, the content of *T. versicolor* ranged from 4 m.% to 24 m.% when grown on birch sawdust and from 43 m.% to 44 m.% when grown on hemp shives [60]. The authors mention that deviations due to impurities are, of course, possible and optimization of the method should be done [60].

Quantitative PCR is a very sensitive method, meaning that slight changes in DNA amounts of the standards can change the relation between Cq and DNA concentration. Although the standards were based on fluorometer measurements that precisely detect dsDNA, deviations of a few percent in concentrations cannot be ruled out [61]. Another potential weakness of DNA-based methods is the variation of DNA content or nuclei distribution/abundance in fungal hyphae [62, 63]. The present study tried to minimize these variations by using similar growth conditions (incubation time and wood rather than glucose-based media as representative cellulosic substrate) and five replicates for obtaining the conversion factor between DNA and mycelium. With that, the standard deviation could be kept reasonably low. Differences in DNA yield between fungal species were comparable to those observed in other studies [64, 65]. As a result, the obtained quantification results are not exact values but can definitely be used to compare fungal growth on different substrates. The determined differences between the fabricated composites were realistic when compared to the visual growth inspection. Overall, the presented quantification method provides a good estimation of the amount of fungal biomass in mycelium composites. The fact that qPCR for fungal biomass quantification is proposed in more and more fields, from mass cultivation to truffle detection, additionally supports this method [34, 35, 59, 66–69].

## Conclusions

A qPCR-based quantification of fungal biomass in mycelium composites was applied and tested as a new way to evaluate the suitability of (ligno-)cellulosic residues for mycelial growth. We demonstrate that this method allows for a realistic assessment of the mycelial biomass and can serve as a reliable method for future studies of fungal growth within biomaterials. Information obtained by this method can improve our understanding of the contribution of mycelium to composite material characteristics and help finding the ideal incubation time for a certain application. As for the fungi tested in this study, the performed comparisons revealed that hyphae of *P. pulmonarius* could not provide as much stability as a similar amount of hyphae of *G. sessile* on the same substrate (e.g. PPGW vs. GSGW)*.* For *T. versicolor*, as little as 7 mg of mycelium per cm^3^ of material could ensure good stability for sugar beet pulp whereas spent brewer’s grains containing 34 mg/cm^3^ were fragile. Quantification of the mycelium content in the composites, therefore, demonstrates how crucial the fungal species, the substrate, and their combination are for the material’s overall stability.

Besides the introduction of a new method, this work demonstrated the possibility of upcycling organic side streams to new biomaterials. Most of the substrates used here have not been considered for mycelium composite fabrication so far but showed potential when combined with the right fungal strain. *G. sessile*, for example, grew well on most of the substrates. Sugar beet pulp, on the other hand, formed stable materials only in combination with *T. versicolor*.

In summary, mycelium composites are a promising new class of environmentally benign materials that can incorporate various biomass side streams. Testing different fungi and substrates, as demonstrated here, can be highly recommended to identify combinations leading to stable products with tailored properties. Furthermore, the quantification of mycelium via qPCR promotes a better understanding of its influence on the material properties.

## Supplementary Information

Additional file 1

## Data Availability

The dataset supporting the conclusions of this article is included within the article and its additional file.
